# Plant immune co-receptors: bridging gaps toward next-generation crop defense

**DOI:** 10.1007/s44154-026-00338-w

**Published:** 2026-09-17

**Authors:** Muhammad Mudasir, Ali Shahzad

**Affiliations:** 1 Department of Crop Sciences and Agroforestry, Faculty of Tropical AgriSciences, Czech University of Life Sciences Prague, Kamýcká 129, Prague-Suchdol, 16500 Czech Republic; 2 Sanya Nanfan Research Institute of Hainan University, Sanya, 572025 China

**Keywords:** Plant immunity, Co-receptors, PTI-ETI crosstalk, LRR-RLKs, ROS/Ca^2^⁺ signaling, Crop resistance

## Abstract

Plant innate immunity is a crucial, multi-layered defense system that protects crops from a wide range of bacterial, fungal, and viral pathogens, all of which pose a significant threat to global food security. Co-receptors function as central molecular modulators that amplify and fine-tune immune signaling. This signaling is initiated by cell-surface pattern-recognition receptors (PRRs) and intracellular nucleotide-binding leucine-rich repeat (NLR) proteins. Critical co-receptors for strong immune signal propagation include the SOMATIC EMBRYOGENESIS RECEPTOR KINASE (SERK) protein family, represented by the versatile BAK1, and the SUPPRESSOR OF BIR1-1 (SOBIR1) adaptor. These components work cooperatively to convert pathogen-derived molecular signals into effective cellular defense mechanisms, including the production of reactive oxygen species (ROS), calcium ion (Ca^2+^) fluxes, and the activation of mitogen-activated protein kinase (MAPK) signaling. However, several major knowledge gaps persist. It remains largely unclear how co-receptors physically and functionally orchestrate cross-talk between the two main tiers of defense, pattern-triggered immunity (PTI) and effector-triggered immunity (ETI). A persistent challenge is understanding how shared co-receptors, such as BAK1, avoid disrupting essential developmental pathways, including brassinosteroid (BL) signaling. Furthermore, the spatial and temporal coordination of downstream events within specific plasma membrane microdomains remains poorly understood. In this review, we integrate recent functional and genetic evidence to explain the multifaceted roles of these co-receptors in immune amplification and signaling integration. We discuss the challenges posed by functional redundancy and signaling specificity, and highlight their transformative potential for engineering broad-spectrum disease resistance in crops through precision breeding and targeted modulation strategies.

## Introduction

Unlike animals, plants lack a centralized immune system and instead rely on the autonomous capacity of each cell to activate innate defenses against pathogens. To facilitate this, plants have evolved a sophisticated, multi-layered surveillance mechanism. This system detects non-self or altered-self molecules through immune receptors localized at the plasma membrane and within intracellular compartments (Pradeu et al. 2024; Sharrock and Sun 2020). The innate immune system of plants is a cornerstone of their defense strategy, providing robust mechanisms to detect and respond to a wide range of microbes (Chen 2025; Kong et al. 2024). Plants initiate immune responses through two interlinked systems: pattern-triggered immunity (PTI) and effector-triggered immunity (ETI) (Nabi et al. 2024). Surface-bound PRRs and internal NLR proteins regulate PTI and ETI, respectively. Both immune layers are reinforced by co-receptor assemblies that ensure signaling precision and systemic stability. The interaction between plants and pathogens is best conceptualized through the “Zigzag” model (Fig. 2b), which describes the continuous evolutionary race between plant detection and pathogen evasion. Plant immunity is governed by the synergistic relationship between PTI and ETI. In this model, co-receptors function as essential molecular bridges. They serve to integrate and amplify signals across both defensive layers (Garagounis et al. 2021).

While PRRs and NLRs are well-studied, the co-receptors that are critical amplifiers of immune signaling are still lacking a unified framework to explain their context-dependent roles (Yasuda et al. 2025). The structural and functional diversity of co-receptors underscores their importance in plant immunity. Leucine-rich repeat receptor-like kinases (LRR-RLKs) and leucine-rich repeat receptor proteins (LRR-RPs) are prominent classes of co-receptors that facilitate ligand recognition and signal transduction (Zönnchen et al. 2022). For example, in Arabidopsis, the co-receptor BAK1 (an LRR-RLK) forms complexes with PRRs such as FLAGELLIN-SENSITIVE2 (FLS2) and EF-Tu receptor (EFR), significantly enhancing their sensitivity to microbial signals (Kong and Ramonell 2023). Similarly, LRR-RPs such as RLP23 depend on co-receptors like SUPPRESSOR OF BIR1-1 (SOBIR1) and BAK1 for their functionality, highlighting the complementary roles of these protein families in plant immunity (Bao et al. 2023).

Despite their essential roles, several challenges hinder a complete understanding of co-receptor function. First, functional redundancy, especially among the SERK family (SERK1, SERK2, SERK3/BAK1, SERK4), complicates the genetic dissection of individual co-receptor roles (Taylor 2022). Many co-receptors function across multiple signaling pathways. For instance, BAK1 participates in both PTI and BL signaling. This dual involvement raises critical questions regarding how plants prevent unintended crosstalk. It also highlights the mystery of how signaling fidelity is maintained (Erickson et al. 2022). Third, the spatial dynamics of co-receptors within membrane microdomains remain poorly understood, limiting insights into the spatial regulation of immune activation (Yu et al. 2024; Zhu et al. 2023).

Addressing these gaps is not only of academic interest but also of practical significance. The growing threats posed by climate change and the emergence of novel pathogens necessitate innovative approaches to enhance crop resilience. Co-receptors represent promising targets for genetic engineering and crop improvement, offering the potential to strengthen innate immunity without compromising growth or yield (Song 2024). This review provides a detailed summary of the roles of co-receptors in plant immunity, focusing on their diversity, interactions, and potential applications in biotechnology. We address research challenges and propose future directions to utilize co-receptors.

## Pathogen recognition and the activation of plant immunity

**Fig. 1:**
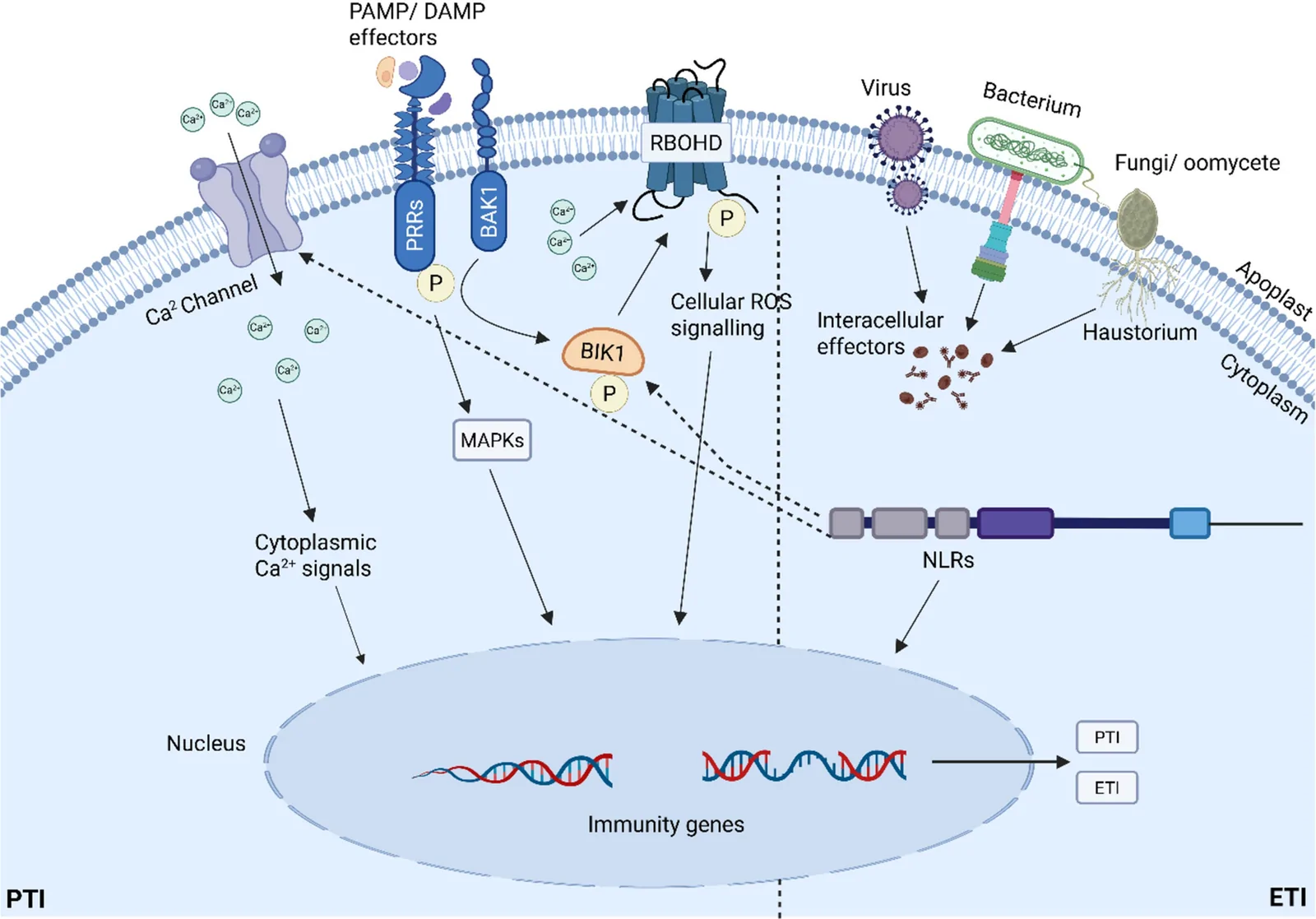
Schematic overview of plant immune signaling pathways involving PRR-mediated PTI and NLR-mediated ETI responses. This figure shows that plant immunity is activated when cell-surface pathogen recognition receptors (PRRs), such as FLS2 and BAK1, detect pathogen-associated molecular patterns (PAMPs) or damage-associated molecular patterns (DAMPs). This recognition event initiates PAMP-triggered immunity (PTI), which in turn activates the MAPK cascade. Intracellular receptor-like cytoplasmic kinases (RLCKs) then phosphorylate key downstream targets, including RBOHD and calcium ion channels (GLRs, CNGCs, OSCA), leading to the production of ROS and an increase in cytoplasmic calcium ion concentration. Intracellular NOD-like receptors (NLRs) detect microbial effectors from bacteria, viruses, fungi, and oomycetes, triggering effector-triggered immunity (ETI). Positive and negative regulatory interactions are represented by arrows and end-blocked lines, respectively. Phosphorylation is indicated by "P," and transcription factors by "TF”

To investigate the roles of receptors, co-receptors, and elicitors in plant defense, a foundational understanding of plant immune systems is required. Plant–microbe interactions have driven the evolution of sophisticated, two-tiered plant immune systems. This conserved system enables plants to recognize potential pathogens through both cell-surface and intracellular surveillance mechanisms (Fig. 1). The first layer of defense involves pattern recognition receptors that detect conserved microbial patterns, while the second layer responds to specific pathogen effectors. These recognition events trigger a cascade of immune responses that are tightly regulated to balance effective defense with energy conservation. The coordinated action of these systems reflects ongoing evolutionary aspects between plants and their pathogens.

### Innate immune pathways

Plant innate immunity is a multi-layered defense system, with the first layer being PTI. PTI is initiated when PRRs recruit essential co-receptors, such as BAK1, transforming a simple recognition event into a robust basal defense response. Without the recruitment of these co-receptors, PRRs remain signaling-silent, highlighting their role as the primary gatekeepers of immune activation (Pradeu et al. 2024; Yu et al. 2024). Upon detection of pathogen effectors by specific resistance (R) proteins, a secondary defense layer is activated, leading to robust ETI. ETI is mediated by intracellular nucleotide-binding leucine-rich repeat (NLR) receptors, which largely constitute the conventional R proteins. These receptors are specialized to detect specific pathogen effectors, often resulting in the induction of a hypersensitive response (Jones et al. 2016; Shah and Gupta 2023).

### PTI-ETI crosstalk: bridging the two layers of plant immunity

**Fig. 2:**
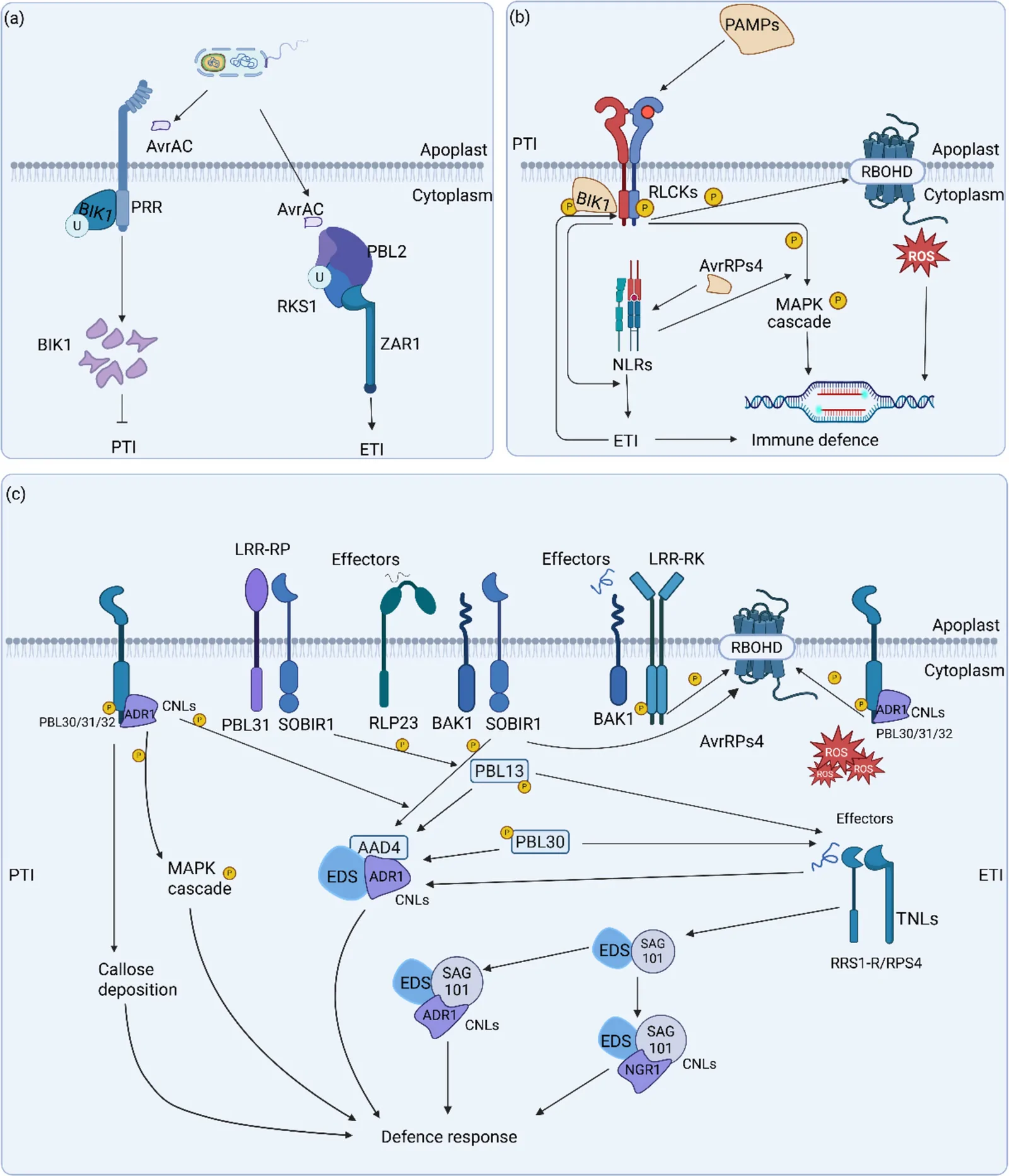
Integration of PRR- and NLR-mediated immune pathways in plants illustrating shared signaling components and crosstalk mechanisms. The crosstalk between PRR- and NLR-mediated immune systems highlights the integrative nature of plant immunity. PRRs, such as LRR-RPs and LRR-RKs, recognize PAMPs to activate PTI, while NLRs detect intracellular effectors to trigger ETI. These pathways share signaling components and synergistically amplify defense responses. For instance, the effector AvrAC uridylates BIK1, suppressing PTI; however, uridylated PBL2, a BIK1 homolog, is detected by RKS1 and ZAR1, thereby activating ETI. Similarly, RLCKs, such as PBL13/30/31/32, interact with helper NLRs (e.g., ADR1 and NRG1) to bridge PTI and ETI signaling. These interactions facilitate ROS production, MAPK cascades, and callose deposition, enhancing plant immunity. The EDS1-PAD4-ADR1 and EDS1-SAG101-NRG1 nodes are critical for integrating PTI and ETI signals. For example, the TNL pair RRS1/RPS4 requires these nodes to activate downstream defense responses upon recognition of the effector. Additionally, PRRs like RLP23 rely on SOBIR1 and RLCKs to form signaling complexes that amplify NLR-mediated ETI. This interconnected network ensures robust immunity by leveraging shared components such as ROS, MAPKs, and callose deposition, which are regulated by both pathways. Thus, PTI and ETI converge at common signaling nodes, enabling plants to mount a coordinated defense against diverse pathogens. Positive and negative regulatory interactions are represented by arrows and end-blocked lines, respectively. Uridylation is indicated by "U," and phosphorylation by "P."

Extensive research has revealed that PRR-mediated immunity and NLR-mediated immunity, though triggered by distinct receptors, are interdependent components of a unified plant defense system. NLR-mediated immunity is often activated in conjunction with PAMP-triggered immunity (PTI) or PTI + ETI, with rare instances of NLR activation in the absence of PTI. The crosstalk between PRRs and NLRs involves synergistic interactions between ROS, Ca^2+^, and phytohormones, leading to the activation of downstream defense genes. This synergistic integration enables the rapid adaptation of the plant to pathogenic challenges while ensuring the optimal allocation of cellular resources. The crucial mechanisms and signaling network governing the PTI-ETI interplay are clearly illustrated in Fig. 2. PTI serves as the frontline defense, directly targeted by pathogen effectors, while NLRs act as a secondary layer, safeguarding PRR signaling pathways. For example, the *Xanthomonas campestris* effector protein AvrAC/XopAC increases bacterial virulence by modifying and degrading the BOTRYTIS-INDUCED KINASE1 (BIK1). This disruption of BIK1 signaling compromises the host's basal immune response during pathogen invasion (Fig. 2) (Wang et al. 2015a, b). Similar to BIK1, the receptor-like cytoplasmic kinase PBL2 is subject to uridylation by the bacterial effector AvrAC. This modification facilitates host recognition and subsequent recruitment of the NLR ZAR1 and RLCK XII RKS1, forming a complex that initiates immune signaling (Wang et al. 2015a, b). Furthermore, a study demonstrates that the CNL ZAR1 can directly perceive the pathogen effector HopZ1a, leveraging the non-functional kinase ZED1 to assemble the ZAR1 resistance complex and activate ETI (Lewis et al. 2013). In Arabidopsis, the NLR RPS5 interacts with the kinase PBS1 to detect the effector AvrPphB, illustrating the integration of PTI and ETI (Carter et al. 2019). The calmodulin-binding protein (CBP60B) serves as a prime illustration of this immunological intersection. Its normal function is to activate transcription of PTI response genes. Specifically, overexpression of CBP60b enhances PTI outputs by upregulating Pathogenesis-Related (PR) gene expression. In contrast, mutations that cause a loss of CBP60B function activate the EDS1-PAD4-ADR1 pathway. The derepression of this module then triggers an autoimmune response mediated by NLR proteins (Li et al. 2021). Thus, CBP60b acts as a molecular rheostat (a biological dimmer switch) that carefully balances immune responses to prevent them from becoming overactive. Additionally, the NLR PigmR protects the ethylene biosynthesis enzyme PICI1 from fungal effector degradation, synchronizing PTI and ETI to ensure disease resistance (Zhai et al. 2022).

### Amplification of immune responses through PTI-ETI crosstalk

The "zigzag" model (Fig. 2b) illustrates the iterative co-evolutionary conflict between pathogen-derived effectors and plant immune receptors, clarifying how PTI and ETI operate as interconnected layers that synergistically enhance the resulting immune outputs (Jones and Dangl 2006; Ngou et al. 2022a, b). For instance, resistance to *Pseudomonas syringae* in Arabidopsis depends primarily on the RRS1-RPS4 NLR protein complex. Research shows that the *rps4rps4b* mutant fails to recognize the AvrRps4 effector. Such observations demonstrate that effective ETI is not independent. Instead, it requires a functional PTI framework to achieve maximum defense efficiency (Ngou et al. 2021). Plant “Helper” NLRs function as calcium-permeable channels, facilitating an increase in cytoplasmic calcium influxes, which results in a rapid and deliberate induction of programmed cell death as a defense mechanism (Jacob et al. 2021). In tomatoes, the helper NLR SINRC4a associates with the PRR LeEIX2, amplifying defense responses triggered by the elicitor EIX (Leibman-Markus et al. 2018). The slnrc4a/slnrc4b double mutant exhibits enhanced resistance, upregulating PRR expression and volatile production (Leibman-Markus et al. 2023).

### Shared signaling pathways in PTI and ETI

Studies indicate a significant degree of interdependence between PTI and ETI signaling pathways (Fig. 2c). Although initiated in different cellular locations, both pathways employ common transcriptional activation mechanisms, suggesting potential upstream integration of nuclear events. For example, the EDS1-PAD4-ADR1 signaling node acts as a central point for defense signaling, activated by both cell-surface RLCPs/RLCKs and intracellular NLRs. This node serves as a crucial integration point for defense signaling pathways, ultimately controlling downstream defense responses (Pruitt et al. 2021). For example, the bacterial effector AvrRps4 activates the NLR pair RRS1-S/RPS4 in Arabidopsis, driving ETI through the EDS1-SAG101 and EDS1-PAD4 modules, which bind to the helper NLRs NRG1 and ADR1, respectively (Dongus and Parker 2021).

Furthermore, SOBIR1 forms a complex with RLCK PBL31, which is essential for PTI signaling. The RLCK proteins PBL30, PBL31, and PBL32 cooperate with RLP23 to recognize nlp20. This recognition triggers several downstream defense responses. These include the generation of reactive oxygen species (ROS), the deposition of callose, and the phosphorylation of MAPKs (Pruitt et al. 2021).

### Co-receptors in PAMP-triggered immunity: molecular interactions and signal transduction

Co-receptors, such as the SERK family, particularly BAK1, form dynamic complexes with PRRs to amplify immune signaling, playing a crucial role in pathogen defense across major crops, including rice, wheat, and maize (Osterhus 2024; Song et al. 2025). The LRRII-RLK subfamily, a subgroup within the RLK superfamily, is recognized as containing essential co-receptors for PRRs. In *A. thaliana*, this subfamily consists of 13 members, which can be categorized into three related clusters: the well-studied SOMATIC EMBRYOGENESIS RECEPTOR KINASES (SERK1-5), a group of LRR-RLKs with currently unknown functions, and the NUCLEAR-SHUTTLE PROTEIN-INTERACTING KINASES (NIK1-3) (Fontes 2024; Sakamoto et al. 2012). SERK3, also known as BAK1, is a widely studied member of the SERK protein family. This protein functions as a crucial co-receptor for various PRRs, including FLS2, EFR, and PEPR1. These PRRs recognize specific PAMPs or damage-associated molecular patterns (DAMPs), amplifying PTI against bacterial and fungal pathogens (Wang et al. 2014; Yu et al. 2024). Once PAMPs are perceived, the FLS2 and EFR receptors form a complex with their co-receptor, BAK1. This interaction is initiated by the presence of a specific pathogen ligand. The resulting rapid phosphorylation activates vital immune pathways. These include the MAPK signaling cascade and the transcriptional control of defense genes. Together, these mechanisms provide a robust defense against pathogenic threats (Chen et al. 2025; Liu et al. 2024; Macho and Zipfel 2014).

Precise regulation of PRR–co-receptor interactions, primarily through modulation of their phosphorylation status, is crucial for activating specific immune responses. For example, in Arabidopsis, the phosphorylation of FLS2 by the co-receptor BAK1 is a key event that enhances FLS2 stability and amplifies its signaling capacity. In contrast, the activity of protein phosphatases, such as PROTEIN PHOSPHATASE 2 A (PP2A), leads to the dephosphorylation of FLS2, effectively attenuating the interaction and dampening the downstream signaling cascade (Rhodes 2019; Zhou et al. 2023). Similarly, in rice, phosphorylation of the PRR CERK1 by *OsSERK2* is essential for chitin-induced immune responses (Pradeu et al. 2024). These regulatory controls ensure precise modulation of immune signaling, preventing excessive activation that would impair plant growth and development. A critical, unresolved question is how a single coreceptor, such as BAK1, initiates divergent outputs, including a ROS burst versus transcriptional reprogramming. While current research focuses on “phosphorylation codes”, an alternative model suggests spatial separation within plasma membrane microdomains. If immune complexes are sequestered into distinct lipid rafts, this physical isolation could prevent interference with signaling. However, the temporal dynamics of these microdomains are still poorly understood, leaving it unclear whether spatial clustering is a cause or a consequence of receptor activation.

### LRR-RLP signaling mechanisms in plant defense

**Fig. 3:**
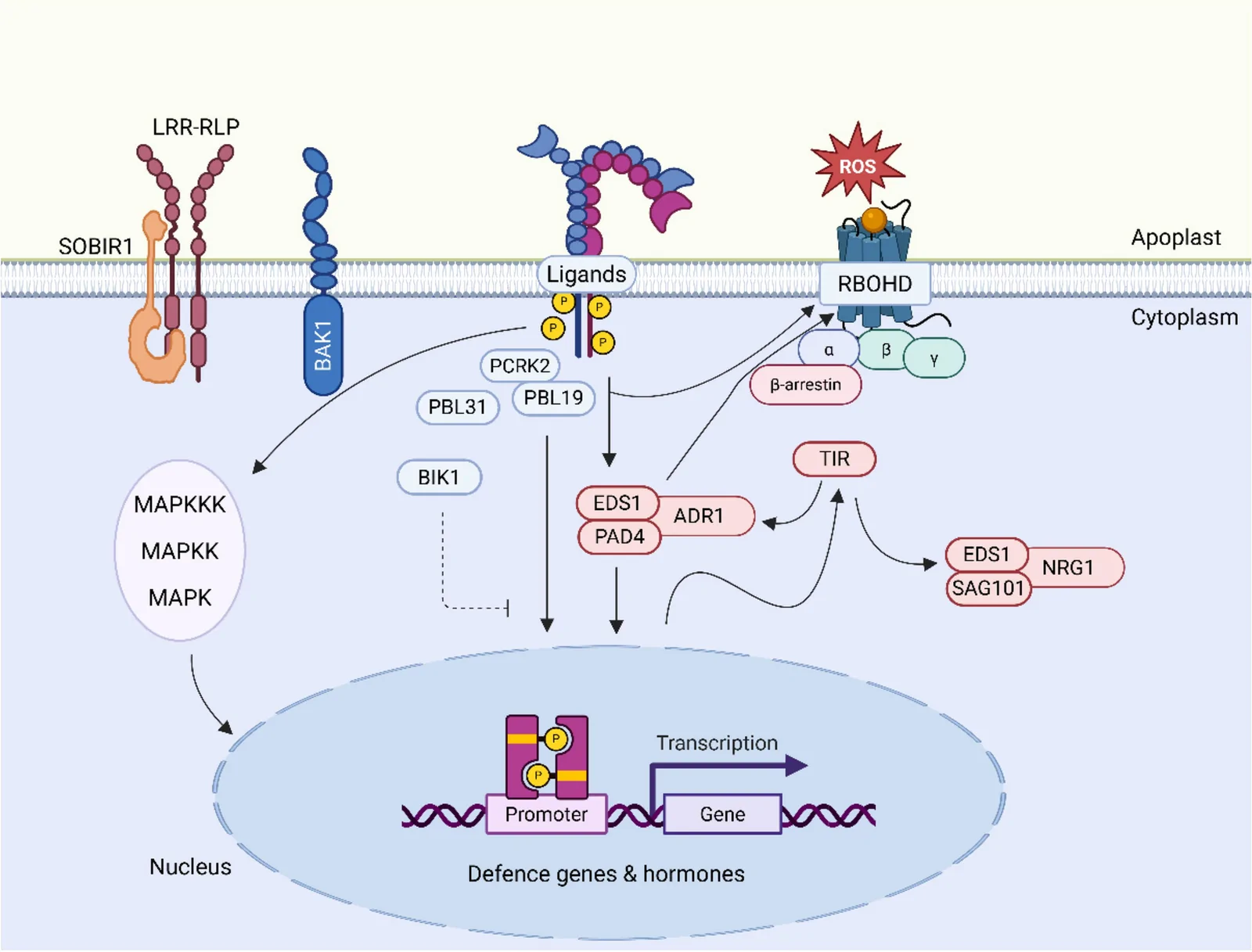
Mechanism of LRR-RLP-triggered immune signaling and downstream activation of defense pathways in plants. LRR-RLP-triggered immune signaling. Cell surface-localized LRR-RLPs are constitutively associated with SOBIR1. Cell surface-initiated immune signaling mediated by LRR-RLPs involves a series of intricate regulatory mechanisms. LRR-RLPs, in constitutive association with SOBIR1, trigger the recruitment of BAK1/SERKs upon PAMP perception, resulting in the activation of SOBIR1 kinases through transphosphorylation. Downstream RLCKs (PCRK2, PBL19, PBL31) are subsequently activated, leading to the phosphorylation of RBOHD/MAPKKK and the initiation of ROS production, MAPK signaling, and transcriptional reprogramming. These receptor complexes also activate the EDS1-PAD4-ADR1 module and TIR genes (Ca^2+^-mediated). Heterotrimeric G-proteins contribute to ROS generation, whereas BIK1 functions as a suppressor of LRR-RLP-induced immune responses through uncharacterized mechanisms

LRR-RLPs function as key pattern recognition receptors in plant immunity, with their extracellular domains detecting PAMPs and initiating defense signaling cascades (Wei et al. 2022). Recognition of PAMPs by LRR-RLPs stimulates the formation of co-receptor complexes. Specifically, the LRR-RLP BAK1 acts as a common co-receptor for various LRR-RLP immune receptors, including RLP32 (Fan et al. 2022). LRR-RLPs lack a cytoplasmic kinase domain. They rely on an interaction with the adaptor protein SOBIR1 to transduce extracellular PAMP signals to intracellular components (Fan et al. 2022; Zhang et al. 2021). The stable association between SOBIR1 and LRR-RLPs is mediated by the GxxxG dimerization motif in the transmembrane region of SOBIR1, occurring independently of PAMP-triggered signaling (Bi et al. 2016; Wei et al. 2022). PAMP-triggered interactions between LRR-RLPs and BAK1 promote reciprocal phosphorylation between SOBIR1 and BAK1, leading to the activation of the SOBIR1 kinase (Huang and Joosten 2025) (Fig. 3).

BIK1, a member of the RLCK-VII-7 subfamily, is phosphorylated by the FLS2-BAK1 complex to positively regulate flg22-triggered plant defense responses (Huang and Joosten 2025; Li et al. 2014). Interestingly, although BIK1 is subject to phosphorylation by nlp20, it exerts a negative regulatory influence on RLP23-dependent signaling (Wan et al. 2019). A recent study demonstrates that other proteins within the RLCKVII-7 family function as positive regulators of immunity elicited by nlp20. Specifically, PATTERN-TRIGGERED IMMUNITY COMPROMISED RECEPTOR-LIKE CYTOPLASMIC KINASE2 (PCRK2) and PBS1-LIKE19 (PBL19) have been shown to interact with SOBIR1, thereby contributing to nlp20-mediated activation of MPK signaling to enhanced resistance against *Hyaloperonospora arabidopsidis* Noco2 (Cai et al. 2024; Tian et al. 2021). Furthermore, the proteins PBL30, PBL31, and PBL32 play a crucial role in the nlp20-induced signaling pathway, which leads to ethylene production and callose deposition in plants (Pruitt et al. 2021). However, the phosphorylation of these RLCKs by the SOBIR1-BAK1 complex remains to be investigated experimentally.

The protein ENHANCED DISEASE SUSCEPTIBILITY1 (EDS1) forms a complex with PHYTOALEXIN DEFICIENT4 (PAD4), leading to the activation of ADR1-type helper NUCLEOTIDE-BINDING DOMAIN LEUCINE-RICH REPEAT (NLR) immune receptors, thereby initiating an immune response that is implicated in TOLL-LIKE, INTERLEUKIN-1 RECEPTOR (TIR) domain-containing NLR-mediated defense pathways (Sun et al. 2021). The peptide nlp20 activates the expression of genes encoding TIR-domain-containing proteins (*TIR* genes) by initiating a signaling cascade that relies on Ca^2+^ as a crucial second messenger. This Ca^2+^-dependent mechanism leads to the transcriptional upregulation of *TIR* genes (Tian et al. 2021). Consequently, it is probable that upon activation, SOBIR1 facilitates the activation of the EDS1-PAD4-ADR1 module through an indirect mechanism involving *TIR* genes. Furthermore, comprehensive biochemical investigations have documented the association of SOBIR1 with PAD4, EDS1, and ADR1, implying direct regulatory control over these elements. Contrasting results were found in another study, which did not detect a connection between SOBIR1, PAD4, and EDS1 (Pruitt et al. 2021; Tian et al. 2021). Therefore, further studies are necessary to clarify how the LRR-RLP/SOBIR1 complex influences the EDS1-PAD4-ADR1 signaling cascade (Fig. 3).

### LRR-RLKs signaling mechanisms in plant defense

**Fig. 4:**
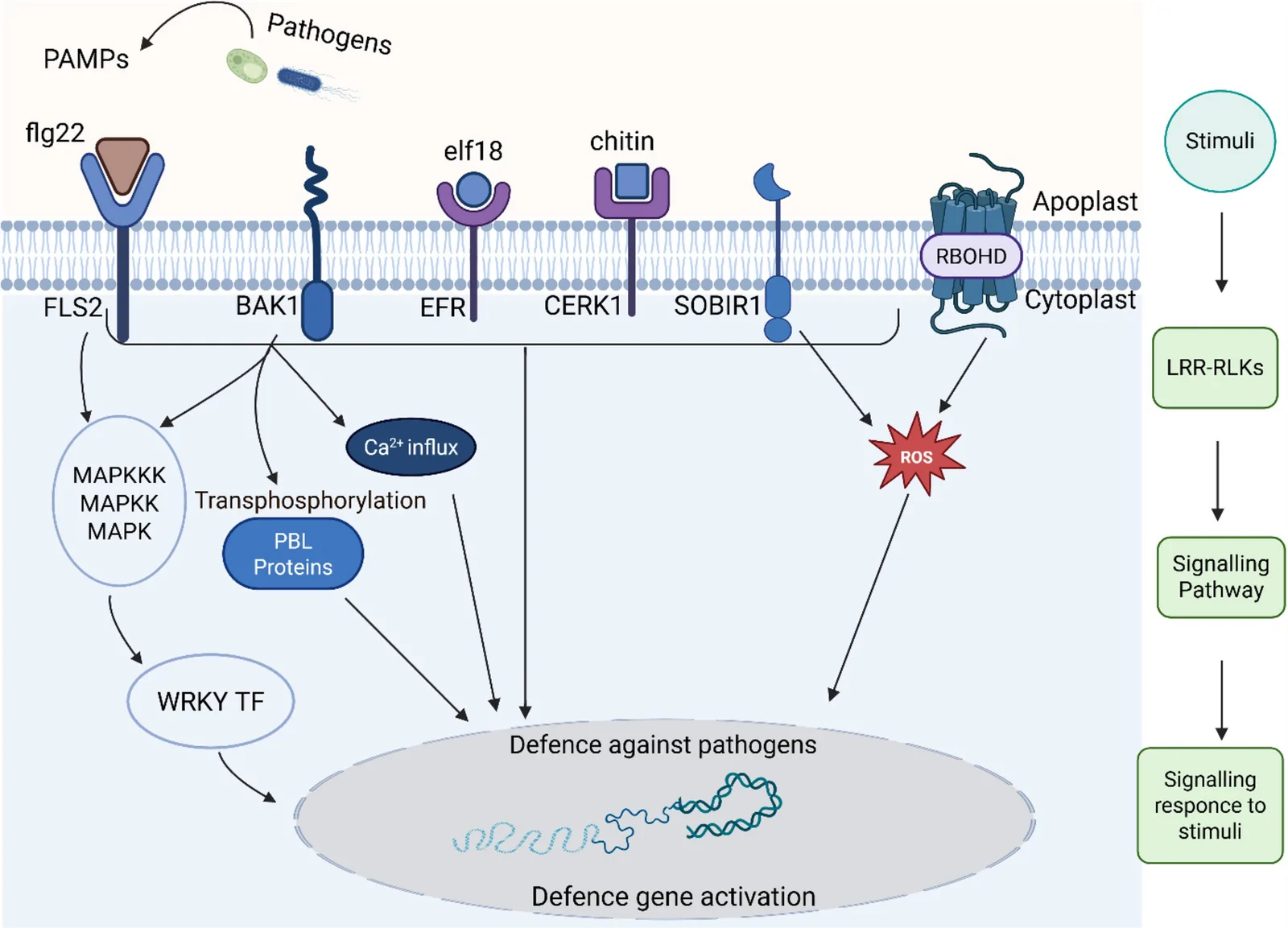
LRR-RLK-triggered immune signaling cascade upon PAMP recognition in plants. The LRR-RLK-mediated plant immunity signaling pathway is initiated when pathogen-associated molecular patterns (PAMPs) such as flg22, elf18, and chitin are recognized by plasma membrane-localized leucine-rich repeat receptor-like kinases (LRR-RLKs), including FLS2, EFR, and CERK1. Upon ligand binding, these pattern recognition receptors rapidly associate with co-receptors such as BAK1 or SOBIR1, forming active receptor complexes that undergo transphosphorylation. This activation triggers LRR-RLKs, notably BIK1 and related PBL proteins, which phosphorylate downstream targets, including RBOHD, to produce reactive oxygen species (ROS). Concurrently, the MAPK cascade (MAPKKK → MAPKK → MAPK) is activated, resulting in the phosphorylation of WRKY transcription factors. These signaling pathways converge to induce nuclear transcriptional reprogramming, resulting in the activation of defense genes, antimicrobial synthesis, callose deposition, and enhanced pathogen immunity

LRR-RLKs are a diverse family of transmembrane proteins that trigger signaling cascades essential for plant development and immune responses (Gandhi and Oelmüller 2023). A subset of LRR-RKs function as sensors for protein-derived immunogenic patterns, initiating immune responses. The plant receptor FLS2 initiates an immune response by directly binding to flg22, a highly conserved 22-amino acid peptide sequence present within the flagellin protein of many bacterial species (Fig. 4) (Monaghan and Zipfel 2012; Ngou et al. 2024). While Arabidopsis FLS2 (AtFLS2) is capable of recognizing the flg22 elicitor from the plant growth-promoting bacterium *Burkholderia phytofirmans*, the FLS2 receptor in *Vitis vinifera* fails to do so (Trdá et al. 2014). While in *Solanaceae*, recognition of the secondary PAMP flgII-28 from certain *Pseudomonas syringae* pathovars occurs independently of SlFLS2, indicating the existence of two distinct flagellin receptors (Chinchilla et al. 2007; Heese et al. 2007). Interestingly, exchanging the cytosolic kinase domains between the two LRR-RLKs did not disrupt flg22-induced signaling (Yaghmaiean 2021). This observation strongly suggests that the formation of the heteromeric complex alone is adequate to facilitate transmembrane signal transduction. The heteromeric complex is the assembly of different proteins, like FLS2 and BAK1, that is more effective than individual proteins acting alone. Recent research shows that the protein BZR helps explain how BL signaling lowers the plant's immune response to flg22 (Aggarwal 2024; Monaghan and Zipfel 2012). BZR induces the expression of WRKY transcription factors, which act as negative regulators of early plant defense responses, thus modulating the delicate balance between developmental processes and immune activation (Yang et al. 2025). The transcription factor BZR has been identified as a key regulator that suppresses early immune responses during BL signaling; the mechanism ensuring co-receptor fidelity remains debated. Two primary models have been proposed: the ‘competition model,’ in which BRI1 and FLS2 physically compete for a limited pool of BAK1, and the ‘phosphorylation code model,’ which suggests that distinct residues on BAK1 are phosphorylated depending on whether it is bound to a developmental or immune receptor (Gao et al. 2019; Lin et al. 2013). Support for the latter proposition comes from emerging research suggesting that unique molecular signatures within the BAK1 kinase domain serve as determinants for subsequent signaling pathways. Despite these findings, it remains uncertain how such signatures are recognized and processed by cytoplasmic kinases, including BIK1. The ability of co-receptors to interact interchangeably with different ligand-binding LRR-RLKs creates significant redundancy, making it challenging to isolate the function of individual LRR-RLK genes using single mutant lines. For example, SERK1, SERK2, and BAK1 co-receptors can all interact with the same ligands, EFR and FLS2. However, FLS2 exhibits a preference for BAK1 over other SERK proteins, whereas EFR does not show a distinct preference for BAK1 (Ding et al. 2024; Roux et al. 2011). The regulatory processes governing the stability of these complexes are largely uncharacterized. For example, the interaction between BAK1 and BIR1 or BIR2 serves to preclude BAK1-FLS2 heterodimer formation, leading to the suppression of immune signaling (Bigeard et al. 2015; Liu et al. 2016). The inherent redundancy within the SERK family (SERK1–4) poses a significant challenge to isolating the functions of individual genes. While BAK1 is the preferred partner for FLS2, other SERKs can partially compensate for its loss, raising the question of whether this redundancy is a ‘fail-safe’ mechanism or a way for the plant to fine-tune responses across different tissue types. This lack of a unified framework for co-receptor ‘preference’ remains a major hurdle in translating these findings into broad-spectrum crop resistance. We have listed some coreceptors, their ligands, and functions in Table 1.

**Table 1:** Co-receptors of LRR-RLP and LRR-RLK with ligands and their functions

RLL-RLPs	Plant species	Ligand	Role	Reference
INR	*Vigna unguiculata* (Cowpea)	inceptins	Resistance to herbivores	(Steinbrenner et al. 2020)
RLP32	*Arabidopsis thaliana*	IF1	Resistance to proteobacterial infection	(Fan et al. 2022)
LeEix2	*Solanum lycopersicum*	Eix	Mediates defense responses by recognizing fungal ethylene-inducing xylanase	(Ron and Avni 2004)
NbEIX2	*Nicotiana tabacum*	EIX-like	Resistance to Verticillium dahlia	(Yin et al. 2021)
ReMax	*Arabidopsis thaliana*	eMax	Early response to mechanical stimuli and jasmonate signaling	(Jehle et al. 2013)
NbRXEG1	*Nicotiana tabacum*	XEG1	Resistance to Phytophthora sojae	(Wang et al. 2018)
RLP17/TMM	*Arabidopsis thaliana*	EPF1, EPF2, Stomagen	Stomata development	(Lin et al. 2017)
LeEix1	*Solanum lycopersicum* (tomato)	Eix	Receptor mediating fungal defense	(Bar et al. 2010)
RLP23	*Arabidopsis thaliana*	nlp20	Resistance to overall plant pathogens	(Albert et al. 2015)
SNC2	*Arabidopsis thaliana*	PSY1	Immune signalling and Peptide growth promoter	(Zhang et al. 2010)
**RLL-RLKs**				
FLS2	*Arabidopsis thaliana*	flg22	Perceives bacterial pathogens and mounts effective immune responses	(Gómez-Gómez and Boller 2000)
PEPR1/2	*Arabidopsis thaliana*	Peps	Amplifying immune responses and linking local and systemic immunity	(Krol et al. 2010)
SERK3	*Arabidopsis thaliana*	flg22, EF-Tu	Enhances immune signaling upon PAMP recognition	(Tang et al. 2017)
SERK4	*Arabidopsis thaliana*	Works with BAK1	Redundantly functions with BAK1 to regulate immune responses	(Tang et al. 2017)
SOBIR1 (EVR)	*Arabidopsis thaliana, Solanum lycopersicum*	RLP-associated	Scaffold protein for RLPs, facilitating defense signaling	(Yin et al. 2002)
CERK1	*Oryza sativa, Arabidopsis thaliana*	Chitin	initiates defense mechanisms against fungal pathogens	(Cai et al. 2024)
LYM1	*Arabidopsis thaliana*	Bacterial peptidoglycan (PGN)	Recognize PGN and activate immune signaling pathways	(Cai et al. 2024)
PSKR1	*Arabidopsis thaliana*	Phytosulfokine (PSK)	regulate cell differentiation and growth	(Yin et al. 2002)
ELR	*Solanum microdontum* (wild potato*)*	Elicitin proteins	activates defense responses	(Cai et al. 2024)
RLP23	*Arabidopsis thaliana*	NLPs	mediates immune responses	(Cai et al. 2024)
Cf-4	*Solanum lycopersicum*	Avr4	Recognizes Avr4 and triggers defense mechanisms	(de la Rosa et al. 2024)
**Other co-receptors**				
LRK10	*Triticum aestivum*	PSY1	Immunity against rust pathogens	(Herrera-Foessel et al. 2012)
NbLRK1	*Nicotiana benthamiana*	Lectin-RLK	Elicitins from Phytophthora species	(Tang et al. 2017)
CEBiP	*Oryza sativa*	Chitin	Forms a complex with OsCERK1 to transduce chitin signaling cascades, leading to immune responses	(Cai et al. 2024)
FER	*Arabidopsis thaliana*	Rapid Alkalinization Factor (RALF) peptides	Serves as a scaffold for PRRs, modulating immune responses	(Tang et al. 2017)
LYK4	*Arabidopsis thaliana*	Chitin, PGN	modulating plant immune responses	(Mittendorf et al. 2024)
ANX1	*Arabidopsis thaliana*	RPS2/RPM1	Negative regulator of both PTI and ETI; interacts with RPS2/RPM1 complexes	(Mang et al. 2017)
ANX2	*Arabidopsis thaliana*	FLS2/BAK1	Negative regulator of both PTI and ETI; interacts with FLS2/BAK1 complexes	(Mang et al. 2017)

### Co-receptors in PTI–ETI crosstalk during oxidative bursts and Ca^2^⁺ signaling

Co-receptors like BAK1 and SOBIR1 coordinate the signaling that results in the formation of ROS/Ca^2^⁺, which is essential for both PTI and ETI. ROS and Ca^2+^ are integral signaling elements that play a crucial role in regulating plant defense mechanisms (Köster et al. 2022). Ca^2^⁺ and ROS are critical secondary messengers that orchestrate robust immune responses; their depletion or impaired signaling can increase plant susceptibility to pathogens. Sensing of pathogens or effectors by cell surface and intracellular receptors triggers biochemical alterations, including Ca^2+^ transients and ROS signaling. These signaling events are instrumental in modulating a repertoire of immune responses (Fig. 1). Co-receptors such as BAK1 and SOBIR1 are the master regulators of these chemical signals; their rapid phosphorylation of downstream targets like RBOHD is the specific trigger for the ROS burst, and Ca^2^⁺ transients that define both PTI and ETIPAMP detection by PRRs activate (Marcec et al. 2019). A crucial regulatory mechanism, termed the ROS-Ca^2+^ interplay, involves a dynamic and self-amplifying feedback loop. This loop is characterized by the reciprocal activation of ROS-sensitive Ca^2+^ channels and Ca^2+^-dependent RBOH proteins, resulting in a sustained and amplified signaling response (Demidchik and Shabala 2018). Earlier research has demonstrated a synergistic relationship between calcium and ROS, resulting in a favorable modulation of plant defense signal transduction (DeFalco and Zipfel 2021; Marcec et al. 2019; Moeder et al. 2019; Negri et al. 2021).

The activation of both PRR and NLR immune pathways elicits demonstrable changes in intracellular Ca^2+^ levels, highlighting the critical function of calcium signaling in these defense responses (Moeder et al. 2019; Wang et al. 2024). Ca^2+^ signaling is produced through the integrated function of transporters and channels, which release internal calcium and import apoplastic calcium. It is significant to emphasize that PTI employs a heterogeneous suite of Ca^2+^ channels, including cyclic nucleotide-gated channels (CNGCs) (Saand et al. 2015), glutamate receptor-like channels (GLRs) (Toyota 2024), and hyperosmolality-induced Ca^2+^ channels (OSCAs) modalities. ETI-associated Ca^2+^ signaling relies on multimeric NLR resistosomes, which are functional oligomeric complexes of activated NLR proteins. These proteins polymerize to form wheel-shaped pores in the plasma membrane, thereby increasing Ca^2+^ permeability. These pores facilitate rapid Ca^2^⁺ influx from the extracellular space, triggering downstream immune responses, including programmed cell death (Jacob et al. 2021).

In plant defense, ROS act as crucial signaling molecules, regulating plant growth and adaptive responses to biotic and abiotic stresses (Rao and Zheng 2025; Shahzad et al. 2025). Among the RBOH proteins, RBOHD is the primary enzyme responsible for ROS production during innate immune responses (Jwa and Hwang 2025). PAMP-triggered cytosolic calcium flux plays a pivotal role in activating RBOHD. Specifically, the transient elevation of cytosolic calcium induces conformational alterations within the N-terminal EF-hand motifs of RBOHD upon PAMP perception. Subsequently, calcium-dependent protein kinase (CPK)-mediated phosphorylation facilitates ROS production by RBOHD (Ali et al. 2024; Weralupitiya et al. 2024). Despite the established role of calcium and CPKs as activators of RBOHD in pattern-triggered immunity, ROS can induce an increase in intracellular calcium concentration and trigger CPK5 activity (Ravi et al. 2023; Zhang et al. 2024). The reciprocal regulation of ROS and Ca^2+^ significantly contributes to the long-range propagation of intercellular ROS and calcium waves. These waves are hypothesized to modulate systemic signaling in response to both biotic and abiotic stress (Gilroy et al. 2016; Mudasir and Shahzad 2025). Future research should investigate calcium-ROS interactions during plant immunity, particularly their regulation by cell wall receptors and apoplastic signals. This will provide new insights into the complexity of plant defense and identify factors influencing calcium- and ROS-mediated immunity, thereby aiding in improving disease resistance.

## Concluding remarks and future perspectives

Plant co-receptors serve as pivotal modulators within immune signaling networks, functioning as adaptable hubs that integrate and potentiate defense mechanisms in both PTI and ETI. These co-receptors form dynamic assemblies with diverse immune receptors, such as FLS2 and SOBIR1, orchestrating downstream pathways that involve phytohormones and ROS-Ca^2^⁺ signaling. Despite significant progress, critical questions remain regarding the specificity of co-receptor interactions, their structural configurations within native membrane contexts, and the extent of functional redundancy. Recent findings highlight the complexity of immune regulation, such as the selective binding of the glycine-18 residue of flg22 to BAK1 and the role of the transcription factor BZR in immune-developmental signaling crosstalk. Nonetheless, translating insights from model plants to agricultural crops remains a key obstacle, limiting the practical exploitation of the co-receptor for crop enhancement.

Future research must prioritize the structural basis of multi-protein complex assembly; while the "Zigzag" model provides a conceptual framework, the precise spatiotemporal coordination of co-receptor recruitment remains elusive. Emerging technologies such as cryo-electron microscopy (cryo-EM) and AlphaFold-multimer modeling offer unprecedented opportunities to resolve the architecture of large-scale "signalosomes" that include elusive adaptors and NLR-integrated domains (Gao et al. 2019; Jeyaraj et al. 2025; Mudasir et al. 2025; Sultan et al. 2026). Furthermore, synthetic biology offers a transformative pathway for receptor engineering. By employing modular design principles and CRISPR/Cas-mediated genome editing, researchers can now develop "designer receptors" with expanded ligand-binding specificities or enhanced recruitment of co-receptors like BAK1 and SOBIR1 (Banerjee 2025). Integrating these structural insights with high-throughput "omics" and machine learning will be critical for predicting immune outcomes in complex field environments. Ultimately, transitioning from fundamental discovery to the deployment of these engineered immune modules will be pivotal for developing next-generation crops with durable, broad-spectrum resistance, thereby ensuring global food security in an era of escalating biotic stresses and climate instability.

## Data Availability

Not applicable.

## Abbreviations

BAK1: Bri1-Associated Kinase 1
BIK1: Botrytis-Induced Kinase1
BRI1: Brassinosteroid Insensitive 1
CERK1: Chitin Elicitor Receptor Kinase 1
CBP60B: Calmodulin-Binding Protein 60b
CNGCs: Cyclic Nucleotide-Gated Channels
CPK: Calcium-Dependent Protein Kinase
DAMPs: Damage-Associated Molecular Patterns
EDS1: Enhanced Disease Susceptibility1
EFR: Ef-Tu Receptor
ETI: Effector-Triggered Immunity
FLS2: Flagellin-Sensitive2
GLRs: Glutamate Receptor-Like Channels
LRR-RLKs: Leucine-Rich Repeat Receptor-Like Kinases
LRR-RPs: Leucine-Rich Repeat Receptor Proteins
MAPK: Mitogen-Activated Protein Kinase
NLR: Nucleotide-Binding Leucine-Rich Repeat
NIK: Nuclear-Shuttle Protein-Interacting Kinases
PAD4: Phytoalexin Deficient4
PAMPs: Pathogen-Associated Molecular Patterns
PBL19: Pbs1-Like19
PCRK2: Pattern-Triggered Immunity Compromised Receptor-Like Cytoplasmic Kinase2
PR: Pathogenesis-Related
RBOHD: Respiratory Burst Oxidase Homolog D
SOBIR: Suppressor of Brassinosteroid Insensitive 1
